# Clinical outcome predictors for metastatic renal cell carcinoma: a retrospective multicenter real-life case series

**DOI:** 10.1186/s12885-024-12572-4

**Published:** 2024-07-05

**Authors:** Mimma Rizzo, Gaetano Pezzicoli, Valentina Tibollo, Andrea Premoli, Silvana Quaglini

**Affiliations:** 1 Medical Oncology Unit, Azienda Ospedaliera Universitaria Consorziale, Policlinico di Bari, Bari, Italy; 2 Department of Interdisciplinary Medicine, University of Bari “Aldo Moro”, Bari, Italy; 3 Laboratory of Informatics and Systems Engineering for Clinical Research, Scientific Clinical Institute Maugeri, Pavia, Italy; 4 Division of Translational Oncology, Scientific Clinical Institute Maugeri (ICS Maugeri), Pavia, Italy; 5 Department of Electrical, Computer and Biomedical Engineering, University of Pavia, Pavia, Italy

**Keywords:** Renal cell carcinoma, Prognosis, Real-world data, Targeted therapy, Immunotherapy

## Abstract

Over the last decades, the therapeutic armamentarium of metastatic renal cell carcinoma (mRCC) has been revolutionized by the advent of tyrosin-kinase inhibitors (TKI), immune-checkpoint inhibitors (ICI), and immune-combinations. RCC is heterogeneous, and even the most used validated prognostic systems, fail to describe its evolution in real-life scenarios. Our aim is to identify potential easily-accessible clinical factors and design a disease course prediction system. Medical records of 453 patients with mRCC receiving sequential systemic therapy in two high-volume oncological centres were reviewed. The Kaplan-Meier method and Cox proportional hazard model were used to estimate and compare survival between groups. As first-line treatment 366 patients received TKI monotherapy and 64 patients received ICI, alone or in combination. The mean number of therapy lines was 2.5. A high Systemic Inflammation Index, a BMI under 25 Kg/m2, the presence of bone metastases before systemic therapy start, age over 65 years at the first diagnosis, non-clear-cell histology and sarcomatoid component were correlated with a worse OS. No significant OS difference was observed between patients receiving combination therapies and those receiving exclusively monotherapies in the treatment sequence. Our relapse prediction system based on pathological stage and histological grade was effective in predicting the time between nephrectomy and systemic treatment. Our multicentric retrospective analysis reveals additional potential prognostic factors for mRCC, not included in current validated prognostic systems, suggests a model for disease course prediction and describes the outcomes of the most common therapeutic strategies currently available.

**Supplementary Information:**

The online version contains supplementary material available at 10.1186/s12885-024-12572-4.

## Background

Renal cell carcinoma (RCC) is the most common type of kidney cancer in adults and its incidence is expected to increase in the coming years [1, 2]. The clear cell histotype (ccRCC) is the most represented, accounting for two-thirds of diagnoses, while the other cases are classified as non-clear cell RCC (nccRCC), an umbrella definition, which comprises many different histologies often endowed by very different prognoses. In 2022, the fifth edition of the World Health Organization (WHO) classification of urogenital tumours (WHO “Blue Book”) [3] was published, which identifies a total of 21 different forms of RCC, including some new molecularly-defined entities.

Almost two thirds of the patients present with localised or locally advanced disease at diagnosis and thus are susceptible to surgical resection, while one-third present with distant metastases at diagnosis. However, even in one-third of resected patients, the disease recurs [4]. The prediction of metastatic recurrence has been addressed many times in the literature and some predictive scores based on clinico-pathological findings have also been developed, such as the UISS score, [5] the Kattan score, [6] the SSIGN score, [7] and, the most widely used, the Leibovich score, in its most recently revised version [8].

Among all cancers, metastatic RCC (mRCC) is one of the few with a robust prognostic algorithm. In fact, at the time of diagnosis of metastatic disease, RCC patients can be stratified into favourable, intermediate, and poor risk categories using the International Metastatic RCC Database Consortium (IMDC) risk model [9]. This prognostic tool uses the interpolation of clinical data (Karnofsy Performance Status, time from diagnosis to systemic treatment) and laboratory data (Hemoglobin, Neutrophil count, Platelet count, Serum Calcium) to predict patients’ survival. This model has been confirmed by evidence over the last decade; its accuracy and reproducibility are well established in first-line targeted therapy [10, 11] and subsequent treatment lines [11, 12] as well as in non-clear-cell mRCC [13].

The clinical management of mRCC has changed radically over the last three decades. Starting from a paradigm of cytokine-based immunotherapy, which yielded relatively modest results in front of relevant toxicity, the systemic treatment of mRCC evolved with the introduction of vascular endothelial growth factor receptor (VEGFR) inhibitors (VEGFR-TKI), mechanistic target of rapamycin inhibitors (mTOR-I), immunotherapy with immune checkpoint inhibitors (ICIs) and, more recently, immune-based combinations. This has led to a measurable improvement: median overall survival for metastatic RCC has increased from less than 1 year in the 1990s to more than 4 years in the recent clinical trials [14].

In this paper, we present a retrospective analysis of 453 mRCC patients that underwent one or more lines of systemic therapy, in two Referral Centres for mRCC, over the last 15 years. In our analysis we exploit the long observation time to describe the response to different therapeutic approaches available over time. Moreover, we aim to identify clinical variables that could improve the prediction of disease course and metastatic disease recurrence.

## Methods

This is a retrospective, analysis that includes patients with a histologically confirmed diagnosis of mRCC who have been treated with systemic therapy at two Italian Health Institutions (Translational Oncology Unit of the Istituti Clinici Scientifici Maugeri in Pavia and Medical Oncology Unit of the University Hospital of Bari). Patients receiving at least one dose of systemic oncologic treatment were included. The collection and analysis of patient-level data for this article was approved by the Ethics Committee of both institutions (“Comitato Etico Indipendente Policlinico di Bari” and “Comitato Etico Istituti Clinici-Scientifici Maugeri”). The deadline for data collection was 30th June 2022.

Patient, tumor, and treatment-related variables were collected through a review of medical records. Demographic data for each patient included age, gender, and race. Patient-specific variables included height, weight, IMDC risk category (i.e., favourable, intermediate, and poor), and vital status as of 30th June 2022 (alive or deceased). Tumor-specific characteristics collected included histology, stage, sarcomatoid component and metastatic sites. Treatment-related variables included nephrectomy, metastasectomy, radiation therapy to any site, number and type of systemic therapies, start and end date of systemic therapies, and neutrophils, lymphocyte ad platelet counts before the first dose of first-line therapy.

The efficacy of systemic therapies was assessed using several variables including best response, progression-free survival (PFS), and overall survival (OS), based on available medical records or censored as of 30th June 2022. The best response to systemic therapies was assessed from medical record review, imaging reports, and treating physician evaluation. PFS was defined as the time between the date of the first dose of systemic therapies and the date of death or radiologically confirmed progression per RECIST 1.1 criteria or physician’s judgement of clinical progression or patient’s refusal to continue systemic treatment. Overall survival (OS) was defined as the time between the first dose of systemic therapies and the date of either death, last known alive, or last follow-up date.

For each patient, the Systemic Inflammation Index (SII) was calculated before the start of the first therapy line, as the product of the number of neutrophils per blood microliter and the number of platelets per blood microliter divided by the number of lymphocytes per blood microliter (as described by Hu et al.) [15].

For patients with surgically radicalized disease whether localised or locally advanced (classified as M0 in the eighth TNM [16] staging if no distant metastases are present) or oligometastatic (M1 NED according to TNM staging [16]: no evidence of disease after metastasectomy), the risk of disease recurrence was calculated. Patients were stratified for disease recurrence risk according to the stratification criteria of KEYNOTE-564 trial, [17] the only registrative trial in the adjuvant setting to date, as follows: low risk (pT2 or lower N0 M0^16^, WHO/ISUP grade 3 or higher [18]), intermediate-high risk (pT2 N0 M0^16^ WHO/ISUP grade 4^18^ or pT3 N0 M0^16^ any WHO/ISUP grade [18]), high risk (pT4 N0 M0^16^ any WHO/ISUP grade [18] or any pT N + M0^16^ any WHO/ISUP grade [18]), M1 NED [16].

Statistical analysis - Descriptive statistics were used to summarise patient characteristics and treatment-related variables. The Kaplan-Meier method and Cox proportional hazard model were used to estimate and compare survival between groups. Multivariate Cox analysis was used to correlate patient- and treatment-related variables to survival (i.e. and OS). Fisher exact test was used to compare proportions. All tests were two-sided and *p*-values of 0.05 or less were considered statistically significant. Statistical analysis was performed using R and Rstudio (“survival” package) [19].

## Results

### Patients’ characteristics

Four hundred fifty-three patients with a histologically confirmed mRCC diagnosis and at least one dose of systemic therapy for metastatic disease were included. The median follow-up time for patients alive on June 30th 2022 was 26.63 months (range 3.1-181.83 months). The median age at first diagnosis of RCC was 55.91 years (range 18.2–83.5 years), most patients were male (*n* = 353, 77.9%) and median BMI was 25.77 Kg/m^2^ (range 17.78–46.98 Kg/m^2^).

The majority of tumors were of clear cell histology (*n* = 360, 79.4%) followed by papillary RCC (*n* = 63, 13.6%), chromophobe RCC (*n* = 12, 2.6%), Microphthalmia Transcription Factor family (MiT) alteration RCC (*n* = 12, 2.6%), unclassified RCC (*n* = 10, 2.2%), collecting duct RCC (*n* = 4, 1.1%). Mixed histotypes were evidenced in 1.7% of cases, while sarcomatoid features were present in 27.5% of patients (*n* = 125).

Metastases were present at the diagnosis in 199 patients (44.0%), while for the other patients the median metastases onset time was 20.73 months (range 3.03-295.33 months). The most common sites of metastasis onset, were lungs (*n* = 317, 69.9%), lymph nodes (*n* = 223, 49.2%), bone (*n* = 110, 24.2%), kidney (*n* = 87, 19.2%), liver (*n* = 80, 17.6%), brain (*n* = 21, 4.6%), adrenal glands (*n* = 18, 3.9%), peritoneum (*n* = 11, 2.4%), muscles (*n* = 8, 1.7%), pancreas (*n* = 8, 1.7%), pleura (*n* = 6, 1.3%).

IMDC risk categories were calculated before the start of the each systemic therapy line. Before first line therapy, 186 patients (41%) were in the intermediate risk class, 155 (34.2%) were in the favorable risk class, and 112 (24.8%) were in the poor risk class. Before second line therapy, 205 patients (60%) were in the intermediate risk class, 88 (25.5%) were in the favorable risk class, and 49 (14.5%) were in the poor risk class. Before third line therapy, 131 patients (64.2%) were in the intermediate risk class, 40 (19.6%) were in the favorable risk class, and 33 (16.2%) were in the poor risk class.

Median Systemic Inflammation Index (SII) was also calculated, and the median value was 930 *10^9^/L (range 103–7731 *10^9^/L) before the start of the first systemic therapy line, 919 *10^9^/L (range 93-6384 *10^9^/L) before the start of the second systemic therapy line, 1043 *10^9^/L (range 102–4749 *10^9^/L) before the start of the third systemic therapy line.

Patient characteristics are summarized in Table 1.

**Table 1:** Patients’ characteristics

Variable	Number of patients (% of the total number = 453)
Age at diagnosis	
Under 50	148 (32.6%)
50–70	257 (56.7%)
Over 70	48 (10.7%)
Gender	
Male	353 (77.9%)
Female	100 (22.1%)
BMI	
< 25 Kg/m^2^	184 (40.6%)
≥ 25 Kg/m^2^	264 (59.4%)
Histology	
Clear Cell	360 (79.4%)
Papillary	63 (13.9%)
Chromophobe	12 (2.6%)
MiT Alteration	12 (2.6%)
Unclassified	10 (2.2%)
Collecting Duct	4 (0.8%)
Stage at first diagnosis	
I-III	254 (56.0%)
IV	199 (44.0%)
Metastatic sites	
Lung	317 (69.9%)
Lymph nodes	233 (49.2%)
Bone	110 (24.2%)
Kidney	87 (19.2%)
Liver	80 (17.6%)
Brain	21 (4.6%)
Adrenal gland	18 (3.9%)
Peritoneum	11 (2.4%)
Muscles	8 (1.7%)
Pancreas	8 (1.7%)
Pleura	6 (1.3%)
Soft tissues	4 (0.8%)
Spleen	2 (0.4%)
Thyroid	2 (0.4%)
Skin	1 (0.2%)
IMDC before first-line therapy	
Favorable	155 (34.2%)
Intermediate	186 (41%)
Poor	112 (24.8%)
IMDC before second-line therapy	
Favorable	88 (25.5%)
Intermediate	205 (60%)
Poor	49 (14.5%)
IMDC before third-line therapy	
Favorable	40 (19.4%)
Intermediate	131 (64.2%)
Poor	33 (16.2%)

*Note*: in the “Histology” section the total is higher than 100%, due to coexistence of more than one single histology in 8 patients

#### Treatments and outcomes

Among the 453 patients, 254 (56.0%) received nephrectomy with curative intent for localized disease, 110 (24.3%) received cytoreductive nephrectomy (in the presence of concomitant distant metastases), 53 (11.7%) received nephrectomy and metastasis resection (M1 NED patients), while 36 metastatic patients (8.0%) did not received nephrectomy. A slightly superior OS was observed in patients receiving cytoreductive nephrectomy versus patients with a metastatic disease onset who did not receive surgery, albeit the result was not statistically significant (28.57 vs. 25.40 months, Cox proportional hazard model *p*-value = 0.18, HR = 0.70, 95%CI = 0.42–1.18).

The mean number of systemic treatment lines received by the 453 patients was 2.5 (range 1–9). Globally, 342 patients (75.4%) received a second-line treatment and 204 patients (45%) reached a third-line treatment. The treatments utilized can be grouped as follows: anti-angiogenic (mainly VEGFR-TKI, but also anti-VEGFR antibodies), immunotherapy (mainly Immune-Checkpoint inhibitors, but also cytokines), anti-angiogenic + immunotherapy combinations, mTOR inhibitors, miscellannea. Broken down data on the different therapies received by the patients in first-line, second-line and third-line, along with the relative outcomes, are reported in Table 2.

**Table 2:** Treatment received by patients, presented along their efficacy outcomes (CR: complete remission; PR: partial remission; SD: stable disease; PD: progression of disease)

Therapy line	Numerosity (%)	Median PFS (months)	CR (%)	PR (%)	SD (%)	PD (%)
**First-line**	**453 (100%)**	**9.01**	**3%**	**26%**	**51%**	**20%**
Anti-angiogenic	366 (80.7%)	9.17	3%	25%	54%	28%
Immunotherapy	16 (3.5%)	12.67	7%	19%	37%	37%
Anti-angiogenic + immunotherapy	48 (10.3%)	18.80	0%	36%	30%	34
mTORi	9 (2.2%)	1.73	0%	20%	30%	50%
Miscellannea	14 (3.3%)	5.53	0%	5%	50%	45%
**Second-line**	**342 (75.4%)**	**5.53**	**1%**	**14%**	**50%**	**35%**
Anti-angiogenic	168 (49.1%)	5.83	1%	19%	50%	30%
Immunotherapy	32 (9.4%)	6.47	0%	15%	60%	25%
Anti-angiogenic + immunotherapy	3 (0.8%)	11.93	33%	0%	66%	0%
mTORi	135 (39.6%)	4.30	1%	7%	52%	40%
Miscellannea	4 (1.2%)	3.48	0%	25%	0%	75%
**Third-line**	**204 (45%)**	**5.27**	**0%**	**13%**	**51%**	**36%**
Anti-angiogenic	117 (57.4%)	8.13	0%	13%	58%	29%
Immunotherapy	18 (8.8%)	3.96	0%	10%	45%	45%
Anti-angiogenic + immunotherapy	3 (1.5%)	8.22	0%	66%	33%	0%
mTORi	60 (29.5%)	3.27	0%	10%	40%	50%
Miscellannea	6 (2.8%)	2.45	0%	0%	17%	83%

The median time between the stop of active therapies and death was 72 days.

More information about the size of each treatment group for each line is reported in Table 2 along with the therapeutic outcomes.

**Fig. 1:**
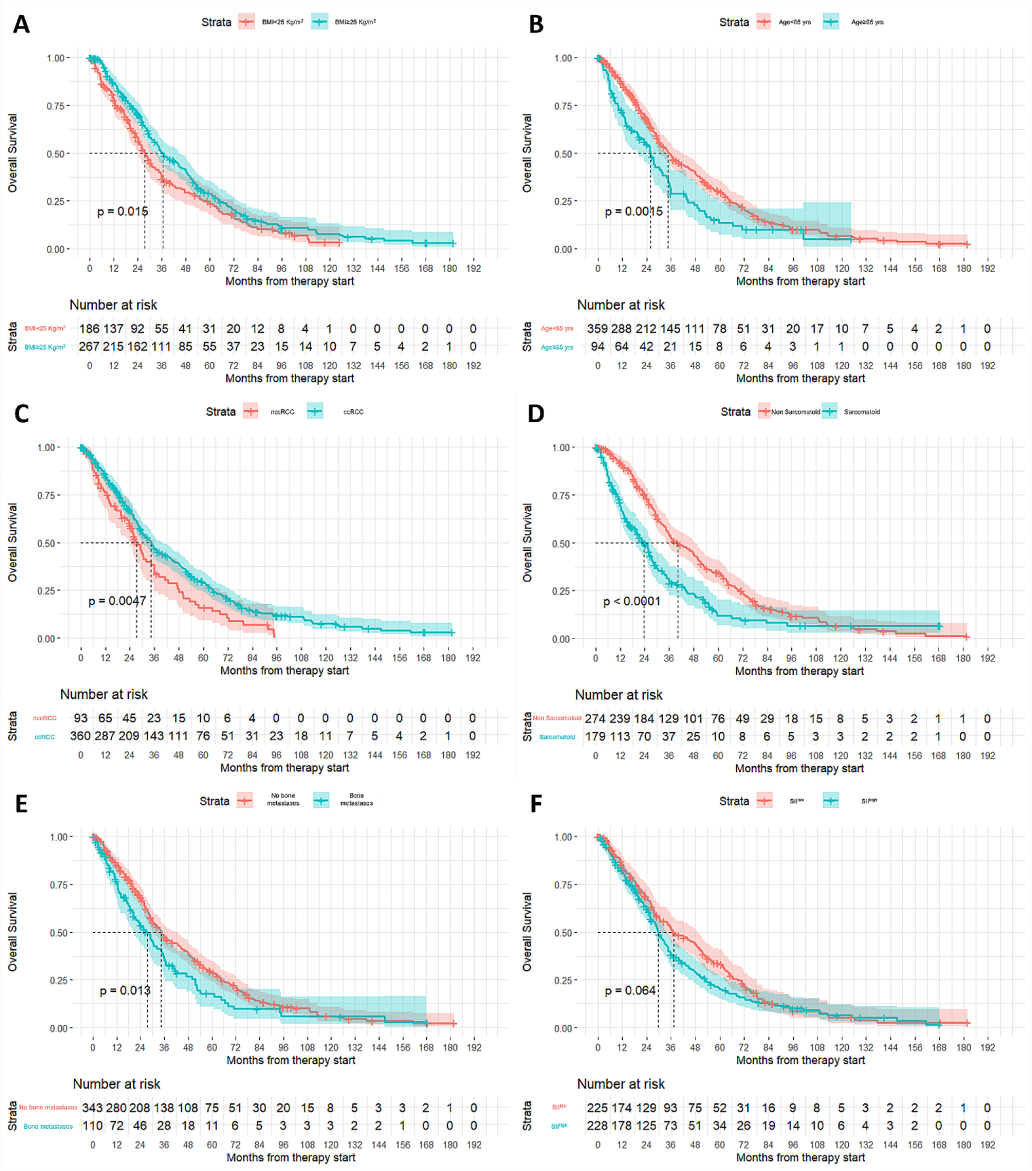
(**A**) Overall Survival probability according to Body Mass Index (BMI). (**B**) Overall Survival probability according to the age at the first diagnosis of RCC. (**C**) Overall Survival probability according to tumor hystotipe. (**D**) Overall Survival probability according to the presence/absence of sarcomatoid component. (**E**) Overall Survival probability according to the presence/absence of bone metastases. (**F**) Overall Survival probability according to the Systemic Inflammation Index (SII)

IMDC score calculated before the start of the first line therapy was confirmed as a good predictor of OS. In fact, the median OS was 64 months for good prognosis patients, 28 months for intermediate prognosis patients and 11 months for poor prognosis patients. The difference was statistically significant (Log Rank *p*-value < 0.0001, Supplementay Fig. 1). IMDC score calculated before the start of the second ant the third line therapy was also significantly correlated with median OS (Log Rank *p*-value < 0.0001). Moreover, the IMDC risk category was significantly associated with the best response, with a higher proportion of favorable-risk patients likely to have experienced disease control (CR + PR + SD) when compared to intermediate and poor-risk patients in first-line therapy (92.2% vs. 71.1%; *p* < 0.0001 by Fisher’s exact test), in second-line therapy (82.2% vs. 53.8%; *p* < 0.0001 by Fisher’s exact test), and in third-line therapy (72.2% vs. 50.9%; *p* < 0.0001 by Fisher’s exact test).

### Factors associated with metastatic disease recurrence

For patients radically resected (both localized and M1 NED), the median time to systemic therapy was 25 months. Their risk of relapse was calculated taking into account pathological stage and grade, as explained in the methods section, dividing the population into low (*n* = 116), intermediate-high (*n* = 110), high risk (*n* = 28) and M1 NED (*n* = 53), as described above. The relapse risk class was found to significantly correlate with the time to systemic treatment as shown in Supplementary Fig. 2 (Log Rank *p*-value < 0.0001). More precisely, while high and intermediate-high risk classes do not show significantly different therapy-free survival *(*16 and 23 months, respectively, *p* = 0.2*)*, they are significantly different for low risk and M1 NED classes (54 and 6.9 months, respectively).

### Factors associated with poor OS

Among patients-related factors, BMI at the start of the first-line therapy and age at diagnosis were associated to OS (Cox proportional hazard model *p*-value = 0.0488 and 0.043, respectively).

Specifically, patients with a BMI lower than 25 Kg/m^2^, namely underweight and normal-weight patients, showed a lower median OS when compared with overweight patients (27 vs. 37 months, Cox proportional hazard model *p*-value = 0.015, HR = 0.77, 95%CI = 0.62–0.95, Fig. 1A). The overall risk of death is higher for underweight patients and decreases progressively until BMI reaches 25 Kg/m^2^, and beyond this point it substantially stabilizes (Supplementary Fig. 3). The data was confirmed both in the male population (26.00 vs. 34.50 months, Cox proportional hazard model *p*-value = 0.0466, HR = 0.78, 95%CI = 0.61–1.01) and female population (30.40 vs. 53.30 months, Cox proportional hazard model *p*-value = 0.0065, HR = 0.52, 95%CI = 0.33–0.83).

About age, patients firstly diagnosed at less than 65-years-old had longer median OS than patients diagnosed at an older age (35 vs. 25 months, Cox proportional hazard model *p*-value = 0.0015, HR = 1.52, 95%CI = 1.17–1.98, Fig. 1B). The overall risk of death start to constantly grow after the age of 55 years at the diagnosis. (Supplementary Fig. 4).

As for histologic characteristics, the non-clear cell histology and the presence of sarcomatoid features were correlated with OS. As expected, patients with a non-clear cell histology showed a shorter OS when compared with patients with clear-cell carcinoma (27 vs. 34 months, Cox proportional hazard model *p*-value = 0.0047, HR = 0.69, 95%CI = 0.53–0.89, Fig. 1C).

Similarly, the presence of a sarcomatoid component was even more strongly correlated with decreased OS (24 vs. 40 months, Cox proportional hazard model *p*-value < 0.0001, HR = 1.77, 95%CI = 1.42–2.21, Fig. 1D).

With regard to disease characteristics, neither the number of metastatic sites at diagnosis nor the presence of brain and liver metastases showed a significant impact on OS (Cox proportional hazard model *p*-value = 0.1323, 0.1599 and 0.0711 respectively). In contrast, the presence of bone metastases was correlated with a shorter OS (34 vs. 27 months, Cox proportional hazard model *p*-value = 0.013, HR = 1.37, 95%CI = 1.07–1.76, Fig. 1E).

In addition, we looked for a laboratory variable that could have an impact on OS. We chose to determine the prognostic value of the Systemic Inflammation Index (SII) in our population, as it could be considered a bonafide representation of the immune system status. Overall SII, calculated at the start of the first-line therapy, showed a correlation with OS (Cox proportional hazard regression *p*-value = 0.002). Patients with SII below the median had a better OS than those with SII above the median (37 vs. 30 months, Cox proportional hazard model *p*-value = 0.064, HR = 1.22, 95%CI = 0.99–1.51, Fig. 1F).

All the above-mentioned analyses are summarized in Table 3.

**Table 3:** *Differences in overall survival associated with the most relevant clinical factors*

Clinical factor	Numerosity (%)	Median PFS (months)	Hazard Ratio (95% CI)	Cox proportional hazard model *p*-value	Stastistical Power at 120 months
BMI < 25 mg/m^2^ BMI ≥ 25 mg/m^2^	186 (41%) 267 (59%)	27 37	0.77 (0.62–0.95)	0.015	0.79
Age at diagnosis < 65 years Age at diagnosis ≥ 65 years	359 (79%) 94 (21%)	35 25	1.52 (1.17–1.98)	0.0015	0.88
Clear cell histology Non-clear cell histology	360 (79%) 93 (21%)	34 27	0.69 (0.53–0.89)	0.0047	0.94
Presence of sarcomatoid freatures Absence of sarcomatoid features	274 (60%) 179 (40%)	24 40	1.77 (1.41–2.21)	< 0.0001	0.99
Presence of bone metastases Absence of bone metastases	110 (24%) 343 (76%)	27 34	1.37 (1.07–1.76)	0.013	0.86
SII above the median SII below the median	228 (51%) 225 (49%)	30 37	1.22 (0.99–1.51)	0.064	0.74

### Multivariate analysis

We performed a Cox multivariate analysis to evaluate the combined impact of IMDC, BMI, age at diagnosis, non-clear cell histology, presence of sarcomatoid features, presence of bone metastases and SII value on the OS. All the variables showed a significant (*p* < 0.05) correlation with OS, except for BMI and age at diagnosis. However, the proportional hazard assumption was not fulfilled for all these mentioned variables, with the exception of non-clear cell histology.

Therefore, we proceeded to perform the Cox multivariate analysis after a pre-stratification based on the IMDC score of each patient. IMDC score was chosen as the stratification variable since it was the variable with the lowest *p*-value in the initial model.

In the stratified models, all variables met the proportional hazard assumption.

For patients classified as IMDC favorable risk (IMDC score = 0), the non-clear cell histology was the only factor that negatively affected OS almost significantly (Cox proportional hazard model *p*-value = 0.07).

For patients classified as IMDC intermediate risk (IMDC score = 1–2), the presence of sarcomatoid features and a high SII value negatively affected OS (Cox proportional hazard model *p*-value = 0.038 and 0.083 respectively).

Finally, for patients classified as IMDC poor risk (IMDC score = 3–6), the presence of sarcomatoid features and BMI under 25 Kg/m2 negatively affected OS with high statistical significance (Cox proportional hazard model *p*-value = 0.011 and 0.001 respectively).

### First-line VEGFR-TKI monotherapy outcomes

In total, 366 patients (80.6%) received a VEGFR-TKI monotherapy as a first-line therapy. The median OS was 29.36 months (95%CI: 27.6–36.5 months, range: 1.8-181.83 months), while first-line treatment PFS was 9.1 months (95%CI: 8.1–13.7 months, range 1.4-152.3 months).

IMDC score calculated before the start of the first line therapy was found to be a predictor of Disease Control Rate (DCR) and OS. In fact, the median OS was 64.3 months (range 19.03-181.83 months, 95%CI = 57.5–72.0) for favourable prognosis patients, 28.3 months (range 2.4-124.83 months, 95%CI = 26.8–30.2) for intermediate prognosis patients and 10 months (range 1.8-41.56 months, 95%CI = 8.1–11.8) for poor prognosis patients (Cox proportional hazard regression *p*-value < 0.0001). Moreover, a higher proportion of favourable risk patients experienced disease control (CR + PR + SD) when compared to intermediate and poor-risk patients in first-line therapy (93.8% vs. 77.0%; *p* < 0.0001 by Fisher’s exact test), in second-line therapy (80.1% vs. 51.3%; *p* < 0.0001 by Fisher’s exact test), and in third-line therapy (73.2% vs. 51.6%; *p* = 0.02 by Fisher’s exact test).

### First-line ICI plus VEGFR-TKI combinations outcomes

In total, 48 patients (10.3%) received an ICI plus VEGFR-TKI combination as a first-line therapy. The overall survival resulted to be longer in the monotherapy patients, compared with those receiving a combination(33 vs. 26 months) although the difference was not statistically-significant (Cox proportional hazard model *p*-value 0.48).

However, when stratified for IMDC risk classes, the data shows that for favorable-risk patients, the median OS is significantly better for the monotherapies group (64 vs. not-assessable months, Cox proportional hazard model *p*-value 0.023). For intermediate-risk patients, the two median OSs are quite similar (26 months for combinations, 28 months for monotherapies, Cox proportional hazard model *p*-value = 0.36). Finally, in the poor-risk patients, the combination therapy correlates with a longer OS almost significantly (15 vs. 10 months, Cox proportional hazard model *p*-value = 0.087).

Interestingly, the survival after the end of the first-line therapy was longer in patients receiving TKI monotherapy when compared to patients receiving an ICI plus VEGFR-TKI combination as a first-line therapy (23 vs. 9 months, Cox proportional hazard model *p*-value 0.098, supplementary Fig. 5). The stratification of post first-line survival based on IMDC risk class was not feasible due to the limited size of the combination therapy group in this setting.

### Treatment resistance

Analyses of treatment resistance were performed on patients with a follow-up of at least six months (*n* = 406), in order to correctly use, in the survival anaysis, a variable defined as *Refractoryness*, which is defined according to progression time within 6 months from therapy start.

The time to progression to the first therapy line was obviously found to be correlated with OS (Cox proportional hazard regression *p*-value < 0.0001). However, it is interesting to report that patients that progressed at the first therapy line within 6 months (*n* = 119) had a notably worse OS than those who progressed after 6 months (22.5 vs. 47 months, Cox proportional hazard model *p*-value < 0.0001, HR = 0.49, 95%CI = 0.34–0.63). Moreover, there was no statistically significant difference between those who progressed in the 3 months or less (*n* = 59) and those who progressed between 3 and 6 months (*n* = 60) from the therapy start (21.3 vs. 23.6 months, Cox proportional hazard model *p*-value = 0.82, HR = 1.04, 95%CI = 0.84–1.24, supplementary Fig. 6).

No significant OS differences have been observed in the early progressor group between patients receiving a VEGFR-TKI (*n* = 98, 34.1%) or a combination of immunotheraphy and VEGFR-TKI (*n* = 8, 47.9%) as a first line therapy (19.4 vs. 14.3 months, Cox proportional hazard model *p*-value = 0.85, HR = 0.92, 95%CI = 0.52–1.44).

## Conclusions

Here we report a cohort of 453 patients with a histologically confirmed mRCC diagnosis who received at least one dose of systemic therapy for metastatic disease between January 1st 2006 and June 30th 2022.

Among the biases influencing this retrospective analysis, the wide interval time of accrual is probably the most impactful one. Indeed, during the total 16 years of observation, many radical changes in the clinical management of RCC occurred, including the introduction of next-generation VEGFR-TKIs and ICIs into the therapeutic armamentarium and the advent of VEGFR-TKI plus ICI combinations. As a consequence, this analysis provides a faithful representation of “older” therapeutic strategies (i.e. VEGFR-TKI monotherapies and ICI monotherapies), while the depiction of more recent ones (i.e. combination regimens) is only partial. Another limitation relies on the fact that both Cancer Centers are high-volume referral centres for the diagnostic-therapeutic management of RCC. Therefore, a considerable part of the patients actively searched for these Cancer Centers and travelled long distances to reach them. This inevitably selects the fittest patients and those with rare clinico-pathological variants of the disease. This explains, for example, the relatively high number of MiT alteration RCC and an over-represented population under 50 years of age. Finally, as already demonstrated [20], patients with mRCC treated at higher-volume facilities have a longer survival than those treated at low-volume facilities, and our analysis seems to support this finding.

Besides the abovementioned limitations, our case series offers good terrain for some considerations.

First of all, in this cohort, out of 453 patients receiving first-line systemic therapy for metastatic RCC, 75.4% received also a second-line treatment and 45% reached the third line. These numbers diverge from literature data, according to which only 42–57% of mRCC patients receive a second-line therapy, [21, 22] and only 13–21% of patients advance beyond second-line therapy [23], although the law of diminishing returns is confirmed also in our series [24]. Moreover, we offer further evidence of the prognostic power of the IMDC score, since it has been shown to correlate with OS [9–12, 25]. However, the median OS we observed for each IMDC risk group was longer than those reported in recent real-world literature [26]: 64.27 months for favorable prognosis patients, 27.6 months for intermediate prognosis patients and 10.6 months for poor prognosis patients [27]. This confirms that the knowledge and experience of the healthcare staff of a high-volume RCC referral centre on the appropriate use and sequencing of systemic therapies may have an impact on treatment outcomes [20]. In a large retrospective real-life case collection [28, 29], it was shown that the physician’s experience in toxicity management, by modulating drug schedule and optimising symptomatic therapies [30, 31], can contribute to an increased PFS and OS.

In our retrospective data collection, we identified some factors, not included in the IMDC score, that are associated with a worse OS: BMI under 25 Kg/m^2^ at the start of the first line treatment, age over 65 years at first diagnosis of RCC, non-clear-cell histology, presence of sarcomatoid component, presence of bone metastases at metastatic disease onset and high SII.

In multivariate analysis, we stratified patients for the IMDC risk group in order to identify those factors that add prognostic information. For favorable risk patients, non-clear-cell histology was the most reliable prognostic factor. This finding induces some considerations. On the one hand, a recent meta-analysis confirms that IO-TKI combinations in the first-line treatment of favorable IMDC risk advanced RCC improve PFS, Objective Response rate and CR, but not OS, compared to sunitinib [32]. According to international guidelines [33, 34], these patients could receive either a VEGFR-TKI monotherapy or a VEGFR-TKI plus ICI combination as first-line treatment. On the other hand, in advanced nccRCC, immuno-combination seems to be associated with better OS than VEGF- and mTOR-targeted therapy [35], although these data need to be confirmed in prospective randomised trials. Thus, it might be interesting to understand how in mixed histology tumors the different proportions of ccRCC and nccRCC influence prognosis. For intermediate risk patients, the prognostic factors were the presence of sarcomatoid component and the high SII. RCC with sarcomatoid features (sRCC) is characterized by mesenchymal dedifferentiation, high biological aggressiveness, and poor prognosis [36]. Recent studies confirm that sarcomatoid dedifferentiation leads to poor response to targeted therapies [37]. However, the sarcomatoid variant has a new treatment standard based on ICI, regardless of IMDC risk group or other clinical variables [38]. The SII has been shown to be a useful prognostic marker for several malignant tumors, including pancreatic, [39] gallbladder, [40] non-small-cell lung, [41] laryngeal cancer, [42] and cholangiocarcinoma [43]. A high SII is independently associated with unfavourable survival outcomes in patients with RCC [44]. Our data highlights its prognostic value. Further research on this index in RCC should be performed, to validate an appropriate cut-off. All the prognostic indicators mentioned above, which notably do not imply additional costs to the clinical routine, could be included in a prognostic model that efficiently expands the IMDC score reach. Finally, for poor risk patients, the prognostic factors were the presence of sarcomatoid component and a BMI over 25 Kg/m^2^. The impact of high BMI as a predictor of better survival for mRCC patients has already been reported, [45] and an explanation based on the role of lipid metabolism in RCC has been proposed [46]. However, our finding that this factor has a particular impact on poor-prognosis patients requires an additional explanation. This data could be related not to the tumour itself, but to tolerance to treatments, VEGFR-TKI in particular. Since weight loss is a major and common adverse event with VEGFR-TKIs, [47] and considering that patients generally receive more VEGFR-TKIs in sequence over the disease course, normal weight and underweight patients will have a greater risk of nutrients depletion and all the complications that follow. Instead, overweight patients are less likely to experience the nutritional complications and therefore tolerate treatments better, achieving better efficacy. This should be taken into account when treating in particular underweight poor-prognosis patients to provide early and appropriate nutritional support.

Another interesting finding concerns RCC patients with brain metastases. In our sample, the presence of brain metastases only slightly affects overall survival, without reaching statistical significance. This is likely because many patients with brain metastases have received brain directed treatments (surgery and/or stereotactic body radiotherapy) and achieved local disease control. This finding is consistent with literature evidence [48] and encourage a more intensive multimodal therapeutic strategy in a multidisciplinary context to improve the survival of patients with brain metastases from RCC [49].

We also evaluated early progressors in our case series. Even though the deleterious impact of early progression on overall survival is well-known, our results underline the biological aggressiveness of RCC refractory to first-line systemic treatment. Patients who progress within 3 to 6 months after the start of first-line VEGFR-TKI monotherapy are intrinsically resistant to angiogenesis blockade and are therefore less likely to respond to subsequent treatments [50–52]. Indeed, since angiogenesis is a key and persistent target and the most studied in mRCC [53, 54], the drugs available for subsequent lines of therapy are mainly anti-angiogenic drugs. Our data demonstrate that this detrimental effect of early progression manifests without a statistically significant difference if the patient progresses in the first or second trimester of treatment.

A further interesting result pertains to VEGFR-TKI plus ICI combinations, the current first-choice treatment option recommended by international guidelines [33, 34] for the majority of patients with metastatic ccRCC. Our data show that receiving a VEGFR-TKI plus ICI combination had no significant impact on OS compared to a TKI monotherapy. Moreover, the survival after the end of the first-line therapy was longer in the patients who received a TKI monotherapy in first-line setting. This evidence goes in the same direction as a recent meta-analysis comparing two different VEGFR-TKI plus ICI combinations (Atezolizumab plus Bevacizumab and Avelumab plus Axitinib) versus VEGFR-TKI monotherapy (Sunitinib) [55]. The combinations were associated with a reduced risk of progression (*p*-value < 0.001, HR = 0.78, 95%CI = 0.69–0.88), but they were not associated with risk of death (*p*-value = 0.11, HR = 0.88, 95%CI = 0.75–1.03).^55^ Another interesting evidence comes from the recently presented long-term follow-up of the CLEAR phase III trial (Lenvatinib plus Pembrolizumab vs. Sunitinib in first-line in RCC patients): the experimental combination showed a median OS comparable with the TKI monotherapy OS (53.3 vs. 54.3 months respectively, *p*-value = 0.04, HR = 0.79, 95%CI 0.63–0.99), with an advantage in the OS rates that progressively decreases (80.4% vs. 69.6% at 24 months, 66.4% vs. 60.2% at 36 months, 55.9% vs. 52.5% at 48 months) [56].

Moreover, It is interesting to examine the different outcomes of combinations in our patients with different IMDC risk scores. Consistent with clinical trials and real-world outcomes, our data show that patients with worse prognosis benefit more from immune combinations, [57] whereas good prognosis patients benefit more from sequential monotherapies [32]. For patients with a favourable IMDC prognosis, sequential monotherapies may be sufficient to maximise quantity and quality of life. Instead, for patients with low or intermediate-low (1-factor) risk of IMDC, immune-combinations should not be considered a ‘one-size-fits-all’ remedy, but to be exploited in patients with close treatment compliance requiring significant early disease control.

A last consideration is about the prediction of time to systemic treatment in patients undergoing radical tumor resection. In the therapeutic management of RCC, several effective metastasis-directed therapies (metastasectomy or ablative radiotherapy) can be used. Therefore, an oligometastatic patient could receive several local treatments and achieve long-term disease control before requiring systemic therapy. The start of systemic therapy represents the turning point in a patient’s clinical history, even more than the onset of metastatic disease. Nevertheless, many tools for the prediction of mRCC metastatic relapse exist, [58] but none to predict the time to systemic treatment. In our analysis, we stratified all the radically resected patients (including M1 NED patients) by using a relapse risk score similar to the one used in the KEYNOTE-564 phase III trial, the only positive adjuvant trial in RCC [17]. Although simple, these criteria based on T stage, N stage, M stage and histological grade, rationally stratified our patients, without overlapping in the Kaplan-Meier curves between the groups. In addition to offering a potential tool for predicting the timing of systemic treatment (even if ad hoc clinical validation is still needed), this finding could offer a basis for debating which RCC patients will benefit most from adjuvant ICI therapy. Our data are likely to support the indication of the KEYNOTE-564 trial, in which clinical benefits were observed for intermediate-high risk, high risk and M1 NED patients.

In conclusion, our analysis comes at a pivotal time in the management of mRCC. The change in the therapeutic paradigm, from monotherapies to combinations, is undoubtedly an important step in the mRCC treatment. However, this is also the ideal moment to look back and learn from past mRCC management strategies. Our data offer a good landscape of what monotherapies gave us and what the setting for combinations might be. Furthermore, we have identified some prognostic factors that can be taken into consideration for future research and that could help the clinician in the prognostic decision-making.

### Electronic supplementary material

Below is the link to the electronic supplementary material.

Supplementary Material 1

## Data Availability

The datasets used and/or analysed during the current study are available from the corresponding author on reasonable request.
