# Supplementary material for: Clinical outcome predictors for metastatic renal cell carcinoma: a retrospective multicenter real-life case series
Source: BMC Cancer. 2024 Jul 5;24:804. doi: 10.1186/s12885-024-12572-4 (PMC11225140; doi:10.1186/s12885-024-12572-4)

**Supplementary Figure 1.** *Overall Survival probability according to the IMDC risk group. Patients with favorable risk are labeled as imdc=0, patients with intermediate risk are labeled as imdc=1, patients with poor risk are labeled as imdc=2.*


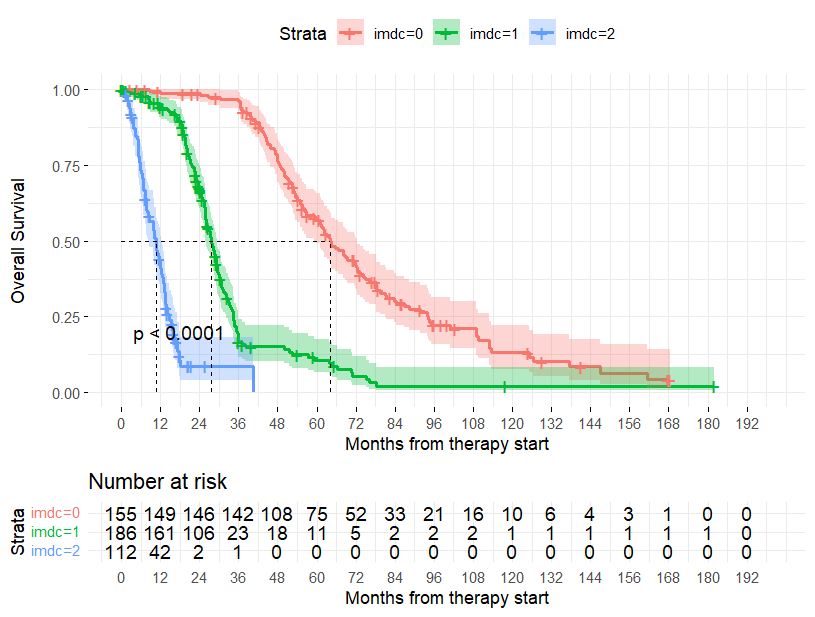


**Supplementary Figure 2.** *Time to Treatment probability according to recurrency-risk class. Patients are divided into low risk, intermediate-high risk, high risk and M1NED, as described in detail in the “Patients and metods” section.*


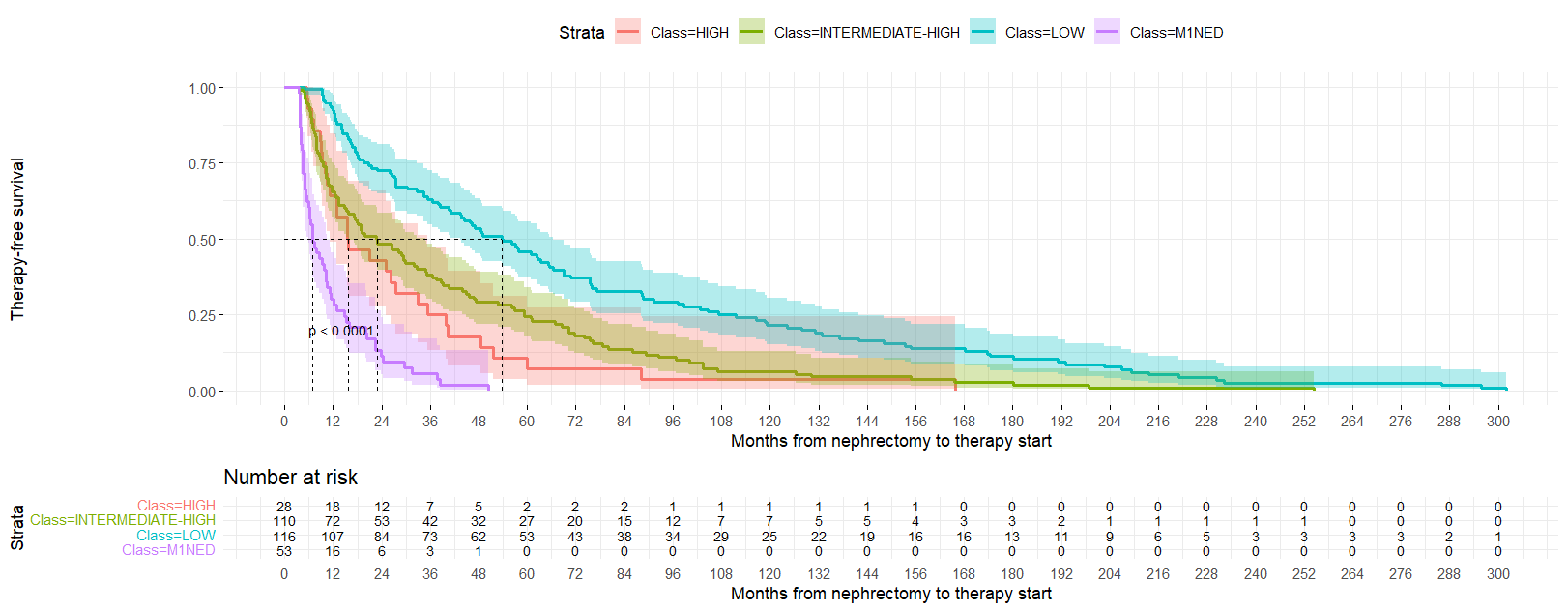


**Supplementary Figure 3.** *Relative hazard of death (log Relative Hazard) in relation to Body Mass Index (BMI), calculated by restricted cubic spline method.*


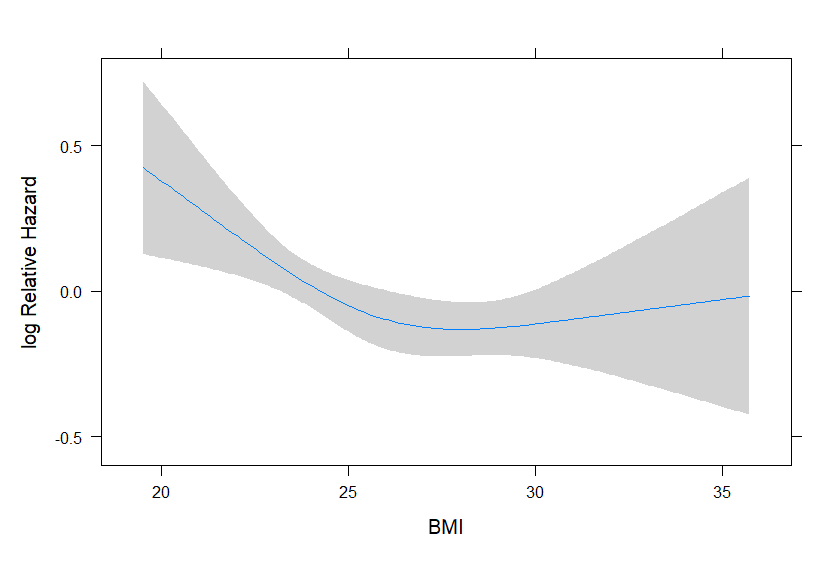


**Supplementary Figure 4.** *Relative hazard of death (log Relative Hazard) in relation to the age at the first diagnosis of RCC, calculated by restricted cubic spline method.*


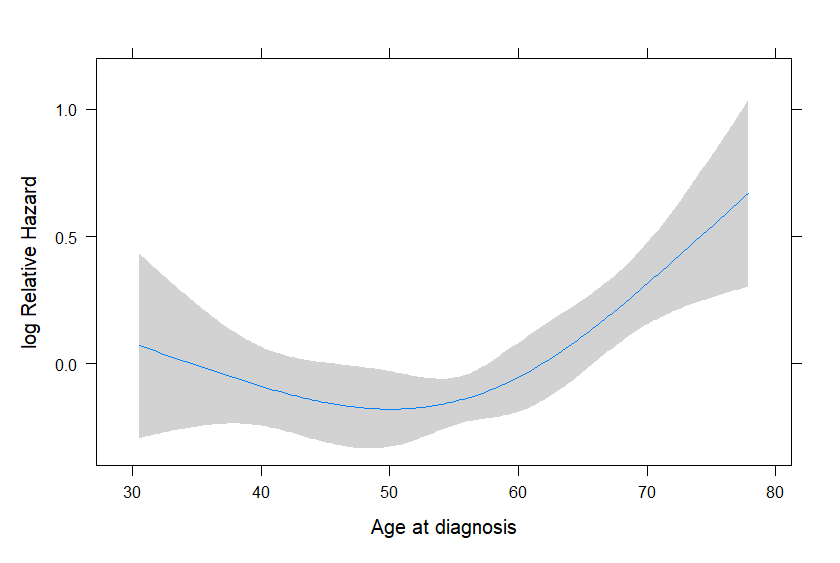


**Supplementary Figure 5.** *Overall Survival probability after the end of first-line treatment in the study population, divided by type of first-line therapy received.*


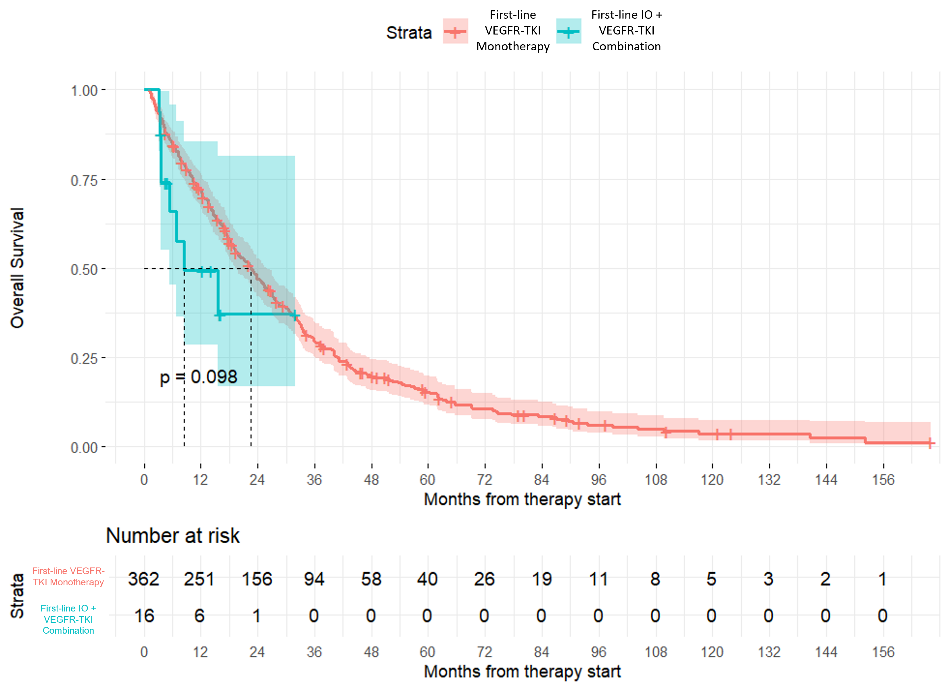


**Supplementary Figure 6.** *Overall Survival probability in the study population, divided by time to first-line systemic therapy failure. Patients who progressed in less than 3 months from therapy start are labelled “Refractoryness=LESS THAN 3 MONTHS”, patients who progressed between 3 and 6 months are labelled “Refractoryness=BETWEEN 3 AND 6 MONTHS” and patients who progressed beyond 6 months from therapy start are labelled “Refractoryness=NON EARLY REFRACTORY”.*


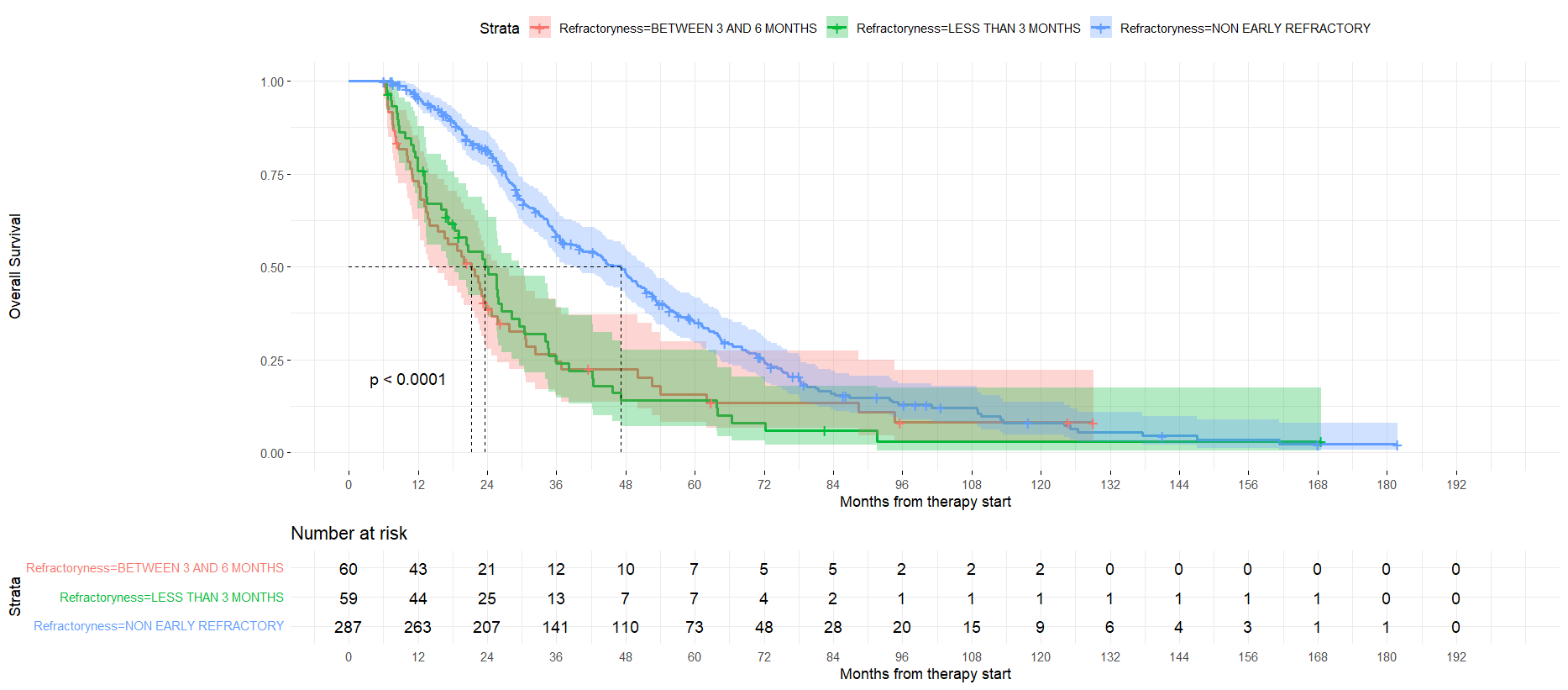

Supplement: Supplementary file 1 — Supplementary Material 1 [file 12885_2024_12572_MOESM1_ESM.docx]
